# Supplementary figures and images for: Baldspot/ELOVL6 is a conserved modifier of disease and the ER stress response
Source: PLoS Genet. 2018 Aug 6;14(8):e1007557. doi: 10.1371/journal.pgen.1007557 (PMC6078684; doi:10.1371/journal.pgen.1007557)

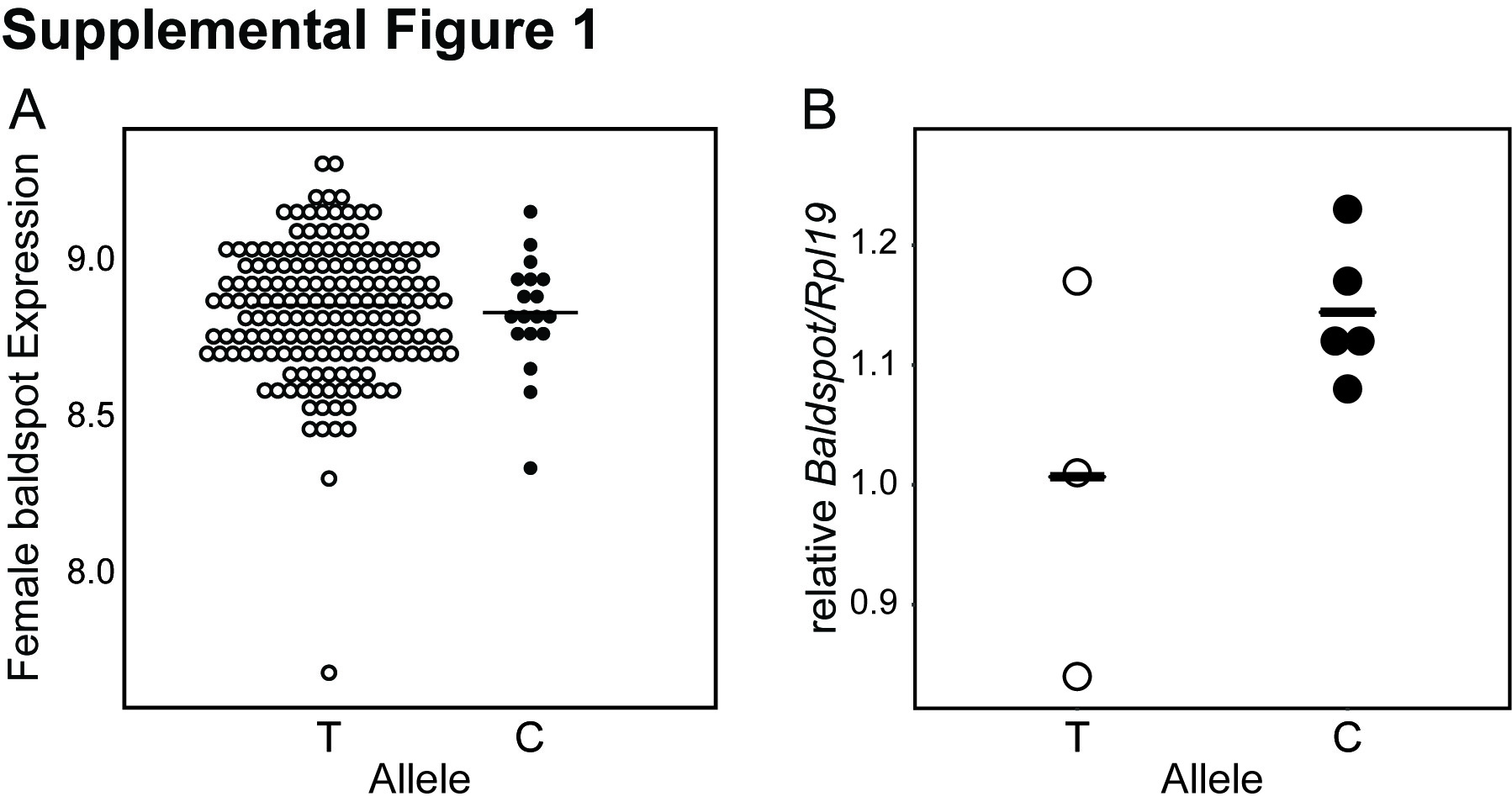

Supplement: S1 Fig — (A) Expression of Baldspot in strains carrying either the T or C allele of the 3L:16644000 SNP was determined from available RNA sequencing data in adult females [56]. Baldspot expression levels was not significantly impacted by this SNP. (B) Expression of Baldspot was not significantly increased in brain-imaginal disc complexes isolated from DGRP lines expressing Rh1G69D and carrying the C allele, as compared to those carrying the T allele. (TIF) [file pgen.1007557.s001.tif]

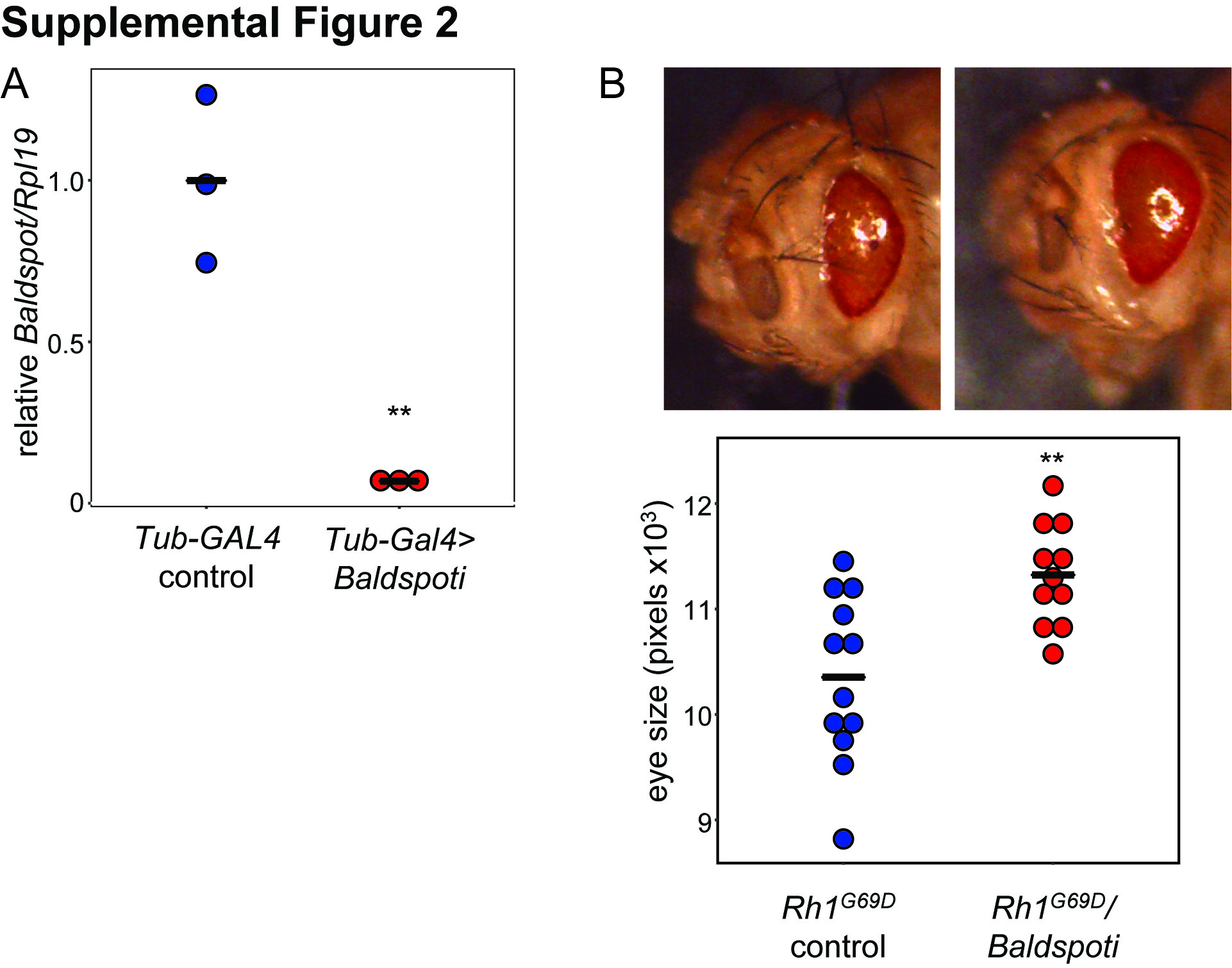

Supplement: S2 Fig — (A) The Bloomington Drosophila Stock Center Baldspot RNAi transgene (44101), used in most of this study, efficiently reduces expression of Baldspot. The RNAi construct was driven ubiquitously by Tubulin-GAL4, and expression determined in wandering L3 larvae. (B) Degeneration caused by overexpression of Rh1G69D is partially rescued by knockdown of Baldspot using a second, independently derived RNAi line from Vienna Drosophila Resource Center (101557KK) (Rh1G69D/Baldspoti vs Rh1G69D controls). Similar to what was observed with the Bloomington Drosophila Stock Center strain, Rh1G69D/Baldspoti flies had larger, less degenerated eyes as compared to Rh1G69D controls (11323 ± 487 pixels vs. controls 10353 ± 798 pixels), indicating that the effects of Baldspot RNAi in this paper are due to loss of Baldspot expression. ** P < 0.005. (TIF) [file pgen.1007557.s002.tif]

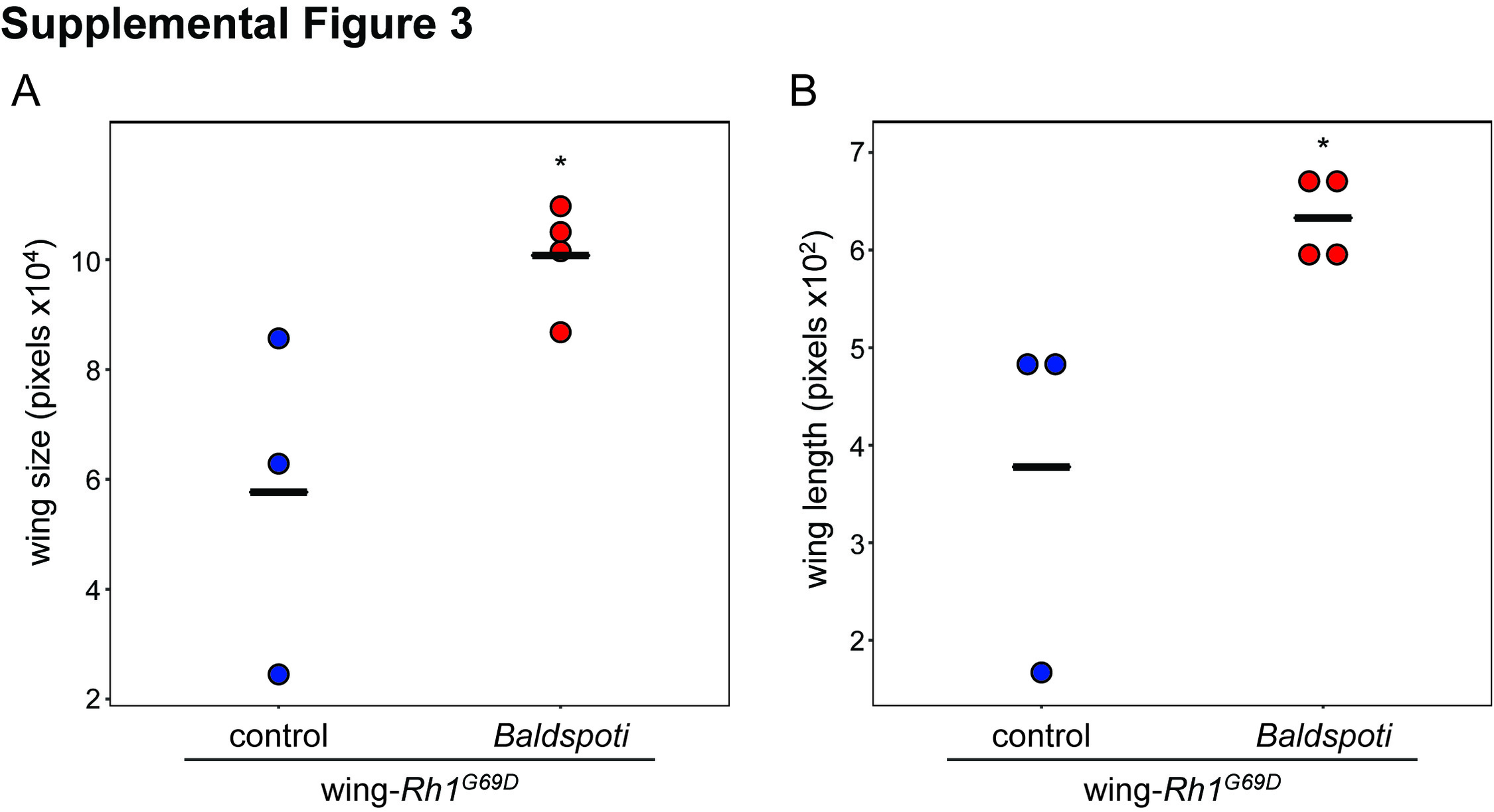

Supplement: S3 Fig — (A) Loss of Baldspot significantly increases the size of wings expressing of Rh1G69D in the wing disc (100769 ± 9903 pixels vs. 57681 ± 30912 pixels in controls). (B) Loss of Baldspot also significantly increases the length of wings expressing of Rh1G69D in the wing disc (633 ± 44 pixels vs. 378 ± 183 pixels in controls). * P < 0.05. (TIF) [file pgen.1007557.s003.tif]

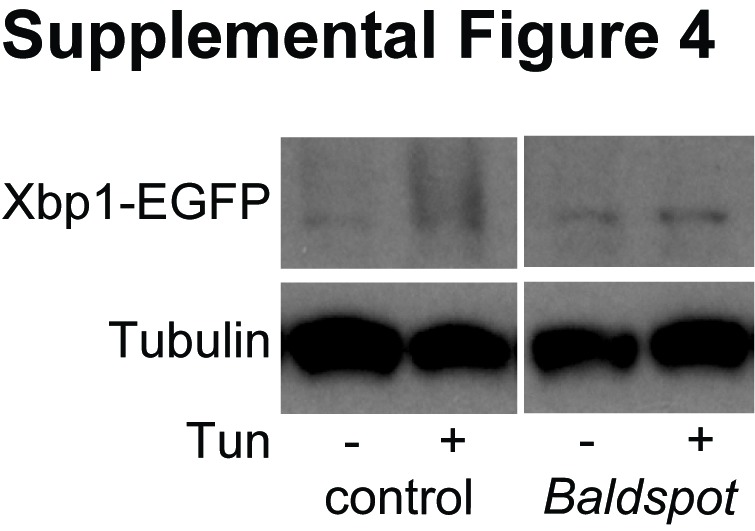

Supplement: S4 Fig — Xbp1-EGFP protein levels are increased in Tub-GAL4 control larvae one hour after treatment with tunicamycin as compared to DMSO-treated controls. Xbp1-EGFP is not increased in Tub-GAL4/Baldspoti larvae. (TIF) [file pgen.1007557.s004.tif]

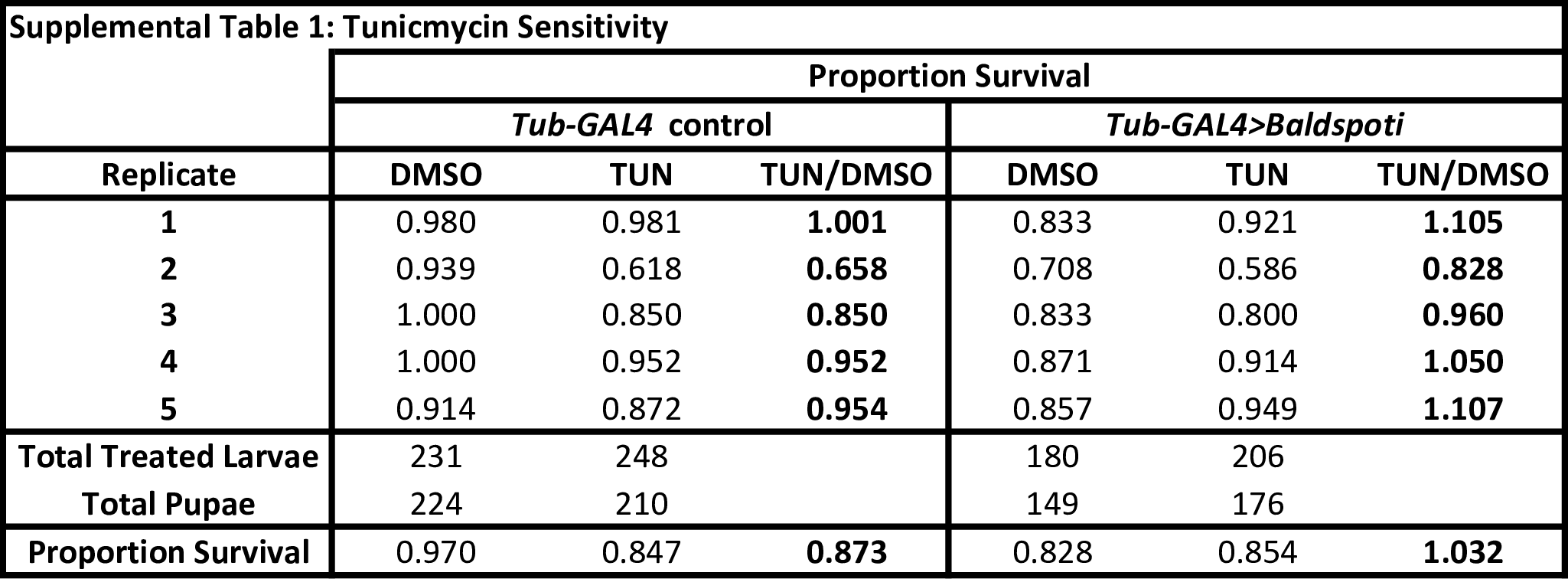

Supplement: S1 Table — (TIF) [file pgen.1007557.s005.tif]
